# Nanofertilizers: A Smart and Sustainable Attribute to Modern Agriculture

**DOI:** 10.3390/plants11192587

**Published:** 2022-09-30

**Authors:** Amilia Nongbet, Awdhesh Kumar Mishra, Yugal Kishore Mohanta, Saurov Mahanta, Manjit Kumar Ray, Maryam Khan, Kwang-Hyun Baek, Ishani Chakrabartty

**Affiliations:** 1 Department of Botany, School of Biological Sciences, University of Science and Technology Meghalaya (USTM), 9th Mile, Techno City, Baridua, Ri-Bhoi 793101, Meghalaya, India; 2 Department of Biotechnology, Yeungnam University, Gyeongsan 38541, Gyeongbuk, Korea; 3 Department of Applied Biology, School of Biological Sciences, University of Science and Technology Meghalaya (USTM), 9th Mile, Techno City, Baridua, Ri-Bhoi 793101, Meghalaya, India; 4 National Institute of Electronics and Information Technology (NIELIT), Guwahati Centre, Guwahati 781008, Assam, India; 5 Department of Biochemistry, Faculty of Life Sciences, Aligarh Muslim University, Aligarh 202002, Uttar Pradesh, India

**Keywords:** agriculture, crop production, nanofertilizer, nano-toxicity, plant growth, sustainability, yield

## Abstract

The widespread use of fertilizers is a result of the increased global demand for food. The commonly used chemical fertilizers may increase plant growth and output, but they have deleterious effects on the soil, the environment, and even human health. Therefore, nanofertilizers are one of the most promising solutions or substitutes for conventional fertilizers. These engineered materials are composed of nanoparticles containing macro- and micronutrients that are delivered to the plant rhizosphere in a regulated manner. In nanofertilizers, the essential minerals and nutrients (such as N, P, K, Fe, and Mn) are bonded alone or in combination with nano-dimensional adsorbents. This review discusses the development of nanotechnology-based smart and efficient agriculture using nanofertilizers that have higher nutritional management, owing to their ability to increase the nutrient uptake efficiency. Additionally, the synthesis and mechanism of action of the nanofertilizers are discussed, along with the different types of fertilizers that are currently available. Furthermore, sustainable agriculture can be realised by the targeted delivery and controlled release of nutrients through the application of nanoscale active substances. This paper emphasises the successful development and safe application of nanotechnology in agriculture; however, certain basic concerns and existing gaps in research need to be addressed and resolved.

## 1. Introduction

Agriculture has evolved in parallel with human evolution. Conventional agriculture demands the regular use of fertilizers, along with traditional agricultural practices, which can tremendously boost the crop growth, the yield, the productivity, and the nutritional value [1]. Hence, chemical fertilizers have played an indispensable role in the growth of modern agricultural practices since the era of the green revolution. In the early years of the previous century, rapid mechanisation occurred in the field of agriculture whereas new technologies, such as marker-assisted breeding and transgenic crop production, were developed in the later years [2]. Although these advancements have helped to increase crop production phenomenally, they exert several harmful effects like diminishing the nutritional quality of soils, decreasing the resistance to pathogens and pests, and exert adverse effects on the environment [3]. Out of the total amount of chemical fertilizers and pesticides applied, more than 50% has been estimated to remain unused as they accumulate in the soil and water bodies through leaching and mineralisation. Owing to the growing awareness of the harmful effects of fertilizers, the last decade has witnessed extensive research into bio-fertilizers, microbiomes, and soil health [4]. By 2050, there will be approximately 9.6 billion people on the planet and this will lead to an increased pressure on the available cultivable land in order to feed the world’s ever-growing population. With the existing advancements and technology, the improvement of crop production and agricultural practices has reached its threshold, i.e., a stage has been reached where it would be nearly impossible to feed the projected population of the near future. Therefore, the development and application of smart agricultural practices using advanced, cutting-edge technologies are urgently needed for sustainable agriculture [5]. This includes the need for the development of new and innovative fertilizers that have a very high efficiency and minimal disadvantages.

Nanotechnology and its associated applications have gained tremendous importance in the present age, as this branch of technology has greatly revolutionised modern science; moreover, this field of science is growing at an exponential rate [6]. Nanomaterials are particles and materials that are handled at a nanoscale range of 1–100 nm. The novel properties of nanomaterials, combined with indigenous and traditional methods, may have tremendous innovative applications in various disciplines of science, including agriculture, which requires innovative methods in order to ensure global food security [7]. The challenge now lies in developing “sustainable and smart” agricultural advancements for rapid crop production. The idea of using nanotechnology in agriculture is not new; several reports that were published by the Department of Agriculture in the USA, Nano-forum, and others have emphasised nanotechnology-based research and application in the agricultural sector [8]. Moreover, nanostructured systems have been used in agriculture for the controlled release of pest-control agents, soil health monitoring, and providing nutrition to the plants. This method is part of the evolving science of precision agriculture, a system that goes hand-in-hand with sustainable agriculture and helps to reduce the energy demand and waste generation, where farmers use technology to efficiently utilise water, fertilizer, and other inputs. In addition, soil quality and fertility also play a crucial role in improving crop quality and yield [9]. Nanofertilizers are nutrients that are encapsulated or coated within nanomaterial in order to enable controlled release, and its subsequent slow diffusion into the soil. The use of nanoscale fertilizers may help to minimise nutrient loss by leaching/run-off and reduce its fast degradation and volatility, thus enhancing the nutrient quality and the fertility of the soil, and promoting crop productivity in the long run [10]. Moreover, because of the high surface area tovolume ratio, and the high penetration ability of nanofertilizers, they may be a suitable alternative to chemical fertilizers. In addition, the use of nanofertilizers, or “nano-biofertilizers”, can reduce the environmental hazard to a large extent. Several reports have revealed that nanofertilizers may boost the crop yield by stimulating seed germination, nitrogen metabolism, photosynthesis, protein and carbohydrate synthesis, and stress tolerance [10]. In addition to the other advantages, these need to be provided to the soil in a relatively lesser quantity, thus enhancing the ease of application and reducing the transportation costs. However, similar to all other fertilizers, the use of nanofertilizers has certain limitations and disadvantages. In this review, we attempt to highlight the need for, and the application of, nanofertilizers for sustainable and smart agriculture [11]. The subsequent sections briefly describe the synthesis of nanofertilizers and the mechanistic approach to how they enhance the soil fertility and improve the crop yield. The advantages of nanofertilizers over the conventional chemical fertilizers have also been discussed, while highlighting the limitations of nanofertilizer application. Lastly, the existing challenges and future prospects for the use of nanofertilizers have been outlined.

## 2. Synthesis of Nanofertilizers

Nanotechnology involves the synthesis and application of devices by managing and controlling their shape and size at the nanometre scale. It has paved the way and enabled the use of nanostructured materials as fertilizers, termed “smart fertilizer” [12]. Additionally, the composition of nanofertilizers can facilitate the efficient nutrient uptake, soil fertility restoration, ultra-high absorption, increased photosynthesis, increased production, reduced soil toxicity, decreased frequency of application, increased plant health, and reduced environmental pollution [13]. Nanomaterial components include silica, Fe, ZnO, titanium dioxide, cerium oxide, aluminium oxide, gold nanorods, ZnCdSe/ZnS core-shell, InP/ZnS core-shell, and Mn/ZnSe quantum dots. The size, the content, the concentration, and the chemical properties of the nanomaterials, in addition to the type of crop, have a significant impact on their effectiveness as nanofertilizers for plant growth. The release of nutrients into the soil occurs when nanoparticle (NP) suspensions containing the nanofertilizers react with water [14]. In order to avoid unfavourable nutrient losses, the nanofertilizers can be coated with polymers or thin coatings in order to encapsulate the NPs.

The efficiency of nutrient use can be improved by applying nanofertilizers that utilise the unique properties of NPs. Nanofertilizers can be produced by adding nutrients individually or in combination to the adsorbents with nano-dimensions. In the case of cationic nutrients, the target nutrients are loaded as is, whereas the anionic nutrients are loaded after surface adjustment to create the nanomaterials using physical and chemical methods [15]. A new technology involves the encapsulation of fertilizers within NPs, which can be accomplished in one of the following three ways: the nutrient can be provided as nanoscale particles or emulsions; it can be coated with a thin polymer layer; or it can be enclosed within nanoporous materials [16]. Controlling matter at 1–100 nm dimensions for use in measurements, creating models for virtual forecasts, and manipulating the nanoscale matter are all included in the definition of a nanofertilizer [17]. Agricultural fields are also affected by solid NPs. The bio-fabrication of NPs using biological processes has received a lot of interest because of the growing need and demand for environmentally friendly, efficient, and non-toxic nanofertilizer synthesis technologies [18]. According to the nutritional requirements for plant growth and development, nanofertilizers can be prepared in three different ways—nanoscale coating fertilizers, nanoscale additive fertilizers, and nanoporous materials [19]. Hydroxyapatite-containing (an important mineral component) nanofertilizers are nutrient delivery systems that are nano-enabled, have a high surface-area-to-volume ratio, and can supply both calcium and phosphorus to plants. Urea-loaded hydroxyapatite nanohybrids are potential nano-encapsulated fertilizers for the gradual release of nitrogen [20]. Owing to the exceptional qualities of mesoporous silica NPs, such as their large surface area, mesoporous architecture, biocompatibility, and nontoxicity, they have the potential to enhance the crop quality and support sustainable agriculture. Silica NPs may directly interact with, and have a positive impact on, plants in several ways, including their effect on plant growth under salinity stress [21]. Carbon-based nanomaterials, such as carbon NPs, carbon nanotubes (CNTs), fullerenes, and fullerols, have become important plant growth regulators, as they increase the rate of germination, the chlorophyll content, and the protein content. Nanofertilizers that are manufactured from organic and inorganic nanomaterials vary depending on the physical or chemical method that is employed. Metal oxides, such as AgO, MgO, ZnO, and TiO_2_, are inorganic nanomaterials, whereas lipids, polymers, and CNTs are organic nanomaterials. Biodegradable, natural, and agriculturally safe carriers, such as chitosan, are used in alternative fertilizer chemicals called polymeric NPs [22]. Owing to the polymeric cationic properties and the ability to interact with negatively charged molecules or polymers, chitosan is a promising agrochemical carrier.

## 3. Mechanistic Approach to How Nanofertilizers Enhance Soil Fertility and Crop Yield

Traditional fertilizers need to be applied in large amounts, as they have low uptake efficiencies. The two main challenges for phosphorus- and nitrogen-based fertilizers are low nutrient uptake efficiency and rapid change into chemical forms that cannot be utilised by plants. This has had a negative impact on the soil and the environment, as the emission of dangerous greenhouse gases and eutrophication have increased [7]. Nanofertilizers gradually release nutrients, which may aid in improving nutrient use efficiency without any related adverse effects. These nanofertilizers are constructed in order to deliver nutrients slowly over an extended time period and to reduce nutrient loss considerably, thereby ensuring environmental safety [22]. Traditional fertilizers are not only costly but may also be harmful to humans and the environment, whereas nanofertilizers play a significant role in maintaining the soil fertility and improving the crop yield [23]. Nanofertilizers enhance plant growth by direct as well as by foliar application methods (Table 1). In order to prevent eutrophication, and to improve nutrient use efficiency in agriculture, nanofertilizers may be the best option [24,25,26]. The organic and inorganic components of the soil can modify the effects of the applied nanofertilizers, depending on their nature and their interactions with the soil. When nanofertilizers are applied to the soil, aggregation occurs first, which reduces the area of action [27], and the aggregates become less mobile in porous materials as their size increases. Hence, the amount of organic matter in the soil, the surrounding environment, and the chemical properties of the nanofertilizers can enhance or reduce the mobility of the NPs [28]. Moreover, nanofertilizers can influence the activity of the soil microorganisms in many ways [29]. Nanofertilizers can increase the nutrient use efficiency, thus being beneficial for nutrition management [4]. These nutrients are bound to the nano-absorbents that are applied either alone or in combination and release the nutrients at a slower rate than that of conventional fertilizers. This approach would minimise nutrient leaching into groundwater and increase the nutrient use efficiency. In order to achieve considerable efficiency in food security, both the agriculture and horticulture sectors are under pressure to utilise alternative fertilizers rather than chemical fertilizers [30]. According to Kale and Gawade [31], in Zn-deficient soil, the addition of ZnO NPs to other fertilizers boosted barley productivity by 91% and improved the nutrient use efficiency when compared to that of the control; however, bulk ZnSO_4_ increased the productivity by only 31% when compared to that of the control. Additionally, nano-composite fertilizers have shown positive effects on rhizosphere bacteria by stimulating the formation of secondary metabolites, which improved plant growth by promoting the colonisation of the root surface [32]. Moreover, research has shown that using “controlled release fertilizer” decreased nitrogen leaching and runoff loss by 25% and 22%, respectively, compared to those by the traditional fertilizers, while also increasing wheat yield and soil residual mineral nitrogen by 6% and 10%, respectively [26,33]. The diverse nanomaterials include silver (Ag), gold (Au), aluminium (Al), single or multi-walled nanotubes, magnetised iron NPs, zinc (Zn), zinc oxide (ZnO), copper (Cu), silica (Si), titanium dioxide (TiO_2_), and cerium oxide (Ce_2_O_3_) [34,35,36]. Owing to the high receptivity of nanomaterials, plants are able to absorb the nutrients and essential chemicals effectively [37]. These “smart fertilizers” are preferable to the conventional fertilizers [38]. Several factors determine the efficiency of nanofertilizers. Both intrinsic and extrinsic factors in crops are highly dependent on the absorption, the transportation, and the accumulation of nanofertilizers, and the route of exposure. The major intrinsic factors determining the efficacy of NPs are the surface coatings and the particle size, whereas the extrinsic factors that strongly affect the potential application of nanofertilizers in crops include the soil texture, the soil pH, and the organic matter [39]. Absorption of nanofertilizers through the roots and leaves considerably influences their behaviour, bioavailability, and the absorption in crops [40]. They control the accessibility of nutrients in the crops by slowing and controlling the release mechanisms, which is advantageous to growers [41]. Nanofertilizers should be synthesised in accordance with the need for the nutrient by the proposed crops, and biosensors should be added to the latest advanced fertilizer, which would control the supply of nutrients according to the requirement of the nutrient in the soil and the condition of the growth period of the plant [42].

The availability of micronutrients during crop growth has a negative effect, as it results in the production of fruits and vegetables with poor nutritional value [53]. NPs can effectively deliver deficient micronutrients, such as Zn and Fe, to crops and fresh food products [53]. Various nanofertilizers (e.g., N, K, P, Zn, Fe, Cu, Mn, Mo, and CNTs) have shown exceptional targeted delivery [54] when they are applied at a specific concentration for diverse crops.

It is known that NPs are present in soil systems [1]. After examining soil in great detail, researchers have discovered a new class of clay particles, termed “Nanosols”, which have a size range of 1–100 nm. De-novo-synthesised NPs exhibit unusual qualities and traits, such as a very high specific surface area (SSAs) and small subsequent surface charges, which cause these NPs to interact with other soil particles vigorously [55]. NPs have a highly resistant nature; they do not degrade quickly and they accumulate slowly in the soil because of their special characteristics [1,56]. The regular use of urea, herbicides, and pesticides is harmful for the fertilisation capacity and the health of the soil [57]. After reaching a certain concentration, urea, herbicides, and pesticides behave similarly to toxic pollutants and become dangerous to both the environment and humans. Moreover, recent studies have suggested that adding NPs to the soil increases the enzymatic activity. Zhao et al. [58] reported that carbon NPs (up to 200 mg kg^−1^) greatly enhanced the activities of urease, dehydrogenase, and phosphatase. They also enhanced the ability of soils to store accessible nutrients, such as ammonium N, nitrate N, and available P [58]. Additionally, Xin et al. reported that urease, phosphatase, and dehydrogenase were stimulated by the impact of multi-walled carbon nanotubes [59]. NPs are of four types, depending on the material, that is, gold, silver, alloy, and magnetic iron oxides, such as Fe_3_O_4_ (magnetite) and Fe_2_O_3_ (maghemite) [60]. Nanofertilizers have been categorised as macronutrient and micronutrient nanofertilizers based on their nutrient content. In addition, nanobiofertilizers (nanomaterials that are combined with microorganisms) are emerging as an alternative approach [54,61].

## 4. Macronutrient Nanofertilizers

Macronutrients, such as N, K, P, Ca, Mg, and S, have been combined with nanomaterials in order to enable the delivery of a precise quantity of these nutrients to crops [4,62]. The macronutrient-based nanofertilizers consist of one or more nutrients that are encapsulated within a particular nanomaterial. The utilisation of NPK in agriculture is likely to increase in the coming years. Thus, research needs to be conducted in order to develop new fertilizers with high nutrient efficiency that are environmentally friendly in order to replace the conventional micronutrient fertilizers.

### 4.1. Nitrogen

Nitrogen (N), which is categorised as a primary macronutrient, is one of the key nutrients; it is deficient in most agricultural soils. Although 78% of N is present in the atmosphere, plants cannot utilise the atmospheric form of N and require nitrogen in the form of nitrates and ammonium. Commercially, it is manufactured using the Haber–Bosch process [63]. Nitrogen fertilizers influence plant growth and development and help to maintain an adequate supply of nutritious food [63]. Due to rapid volatilisation and leaching, these commercially available nanofertilizers (urea) vanish after approximately 75% application. Therefore, the disadvantages of nitrogen fertilizers are their low utilisation and the loss of N to the environment, which contributes to eutrophication and high levels of greenhouse gas emissions [64]. Recently, an advanced N nanofertilizer has been developed using urea-coated hydroxyapatite NPs (rod-shaped structures with an average aspect ratio 10) for targeted delivery and slow release [65]. These compound NPs were selected because of their rich N and P sources, as well as their chemical compatibility.

### 4.2. Phosphorus

**Figure 1:**
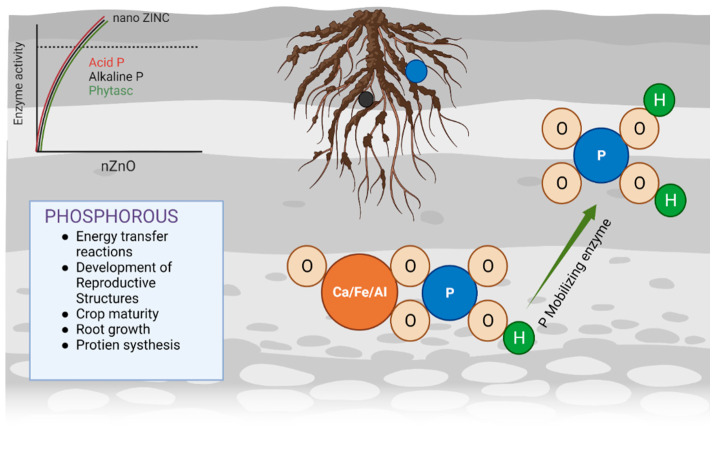
ZnO nanoparticle (NP) treatment increases the activity of P-mobilising enzymes. Consequently, plants uptake Zn ions, and native P mobilisation occurs in the rhizosphere.

Similar to N, P is also fundamental to all living organisms and 90% P is used for food production. Rock phosphate is the raw material for manufacturing most commercial phosphate fertilizers. The major drawbacks of P fertilizers are that they rapidly change from the utilisable form of P into the less accessible forms, they have a low usability and uptake efficiency, and they cause eutrophication (increased N and P levels in water) [66]. Previously, different biological approaches for enhancing P uptake by plants, such as phosphatase and phytase-producing bacterial species and fungi, P-solubilising bacteria, and vesicular–arbuscular mycorrhiza, have been investigated. However, P-mobilising microorganisms were not beneficial because of the low soil organic carbon, limiting the energy supply to the P-mobilising microorganisms, and the high evaporation of water from the soil surface. These factors negatively affect the survivability of the microorganisms [67]. Nanotechnology researchers are investigating methods that will improve the slow release and uptake efficiency of P fertilizers, maintain P in its usable form in the long-term, and allow the mobilisation of native P to plants. Tarafdar et al. [68] used tricalcium phosphate as a precursor salt in order to synthesise fungal-mediated P NPs. Subsequently, Liu and Lal [30] investigated the effect of carboxymethyl cellulose-stabilised hydroxyapatite NPs on soybeans. P nanofertilizer showed promising results for seed treatment when natural raw phosphorite was applied using ultrasonic dispersion [69]. Although P is often present in adequate amounts in the soil, the demand for P fertilizers by farmers is high because the amount of P is not stable for the plants. Zinc NPs were used in order to enhance the efficiency of the conversion of native P into plant-available P. Enzymes such as phosphatase and phytase require Zn as a cofactor. The addition of ZnO NPs increased the activities of these enzymes and P uptake by 11% in legumes and cereals (Figure 1) [36], which also improved the plant growth, the yield, the biomass, and the nutritional significance of cereals and legumes [7]. P-supplements, along with ZnO NP exposure, have also been observed in cotton plants [70]. In cotton, this combination of ZnO supplemented with P increased the biomass, the proteins, and the photosynthetic pigments, which protect against oxidative damage [71]. In order to improve P uptake, native P mobilisation is stimulated by either micronutrient-based NPs (for instance, Fe and Zn) or by generating P-based NPs. This will not only improve the plant growth but also help to address other issues, such as the ecological impacts of P-induced eutrophication and the limited P availability.

## 5. Micronutrient Nanofertilizers

The elements that are necessary for the plant in trace/low quantities are called micronutrients. They are vital for plants to maintain metabolic processes [69]. Zinc (Zn) is a structural or regulatory cofactor for various enzymes and proteins that is required for plant growth [72]. Zn is involved in defence responses against harmful pathogens, the regulation of auxins, the synthesis of carbohydrates, and the protein metabolism [73]. Boron (B) is not only involved in the biosynthesis of the cell wall and its lignification in plants, but it also plays a vital role in plant growth and other physiological processes [74]. Therefore, the application of appropriate amounts of Zn and B is essential for achieving maximum production with high-quality horticultural crops. It was observed that the application of B or Zn nanofertilizers (at three different concentrations) improved the fruit yield (30%) in pomegranate trees (*Punica gratatum* cv. Ardestani) [75]. The shoot and fruit yield were increased when rubber-type nanomaterials were used as a zinc source, compared with the usage of commercial Zn-sulphate fertilizer [76]. Similar results were reported with a Zn NP-related nanofertilizer, which considerably improved the grain yield (38%) of pearl millet (*Pennisetum americanum*). In addition, they were also related to the increase in the length of the root and shoot, the root area, the chlorophyll content, the total soluble leaf protein, and the plant dry biomass, compared to those of the control in 6-week-old plants [77]. A considerable increase in yield was also observed in sunflower, sugarcane, potato, rice, maize, and wheat using Zn NPs as a nutrient source [78]. Moreover, stabilised maghemite NPs enhanced the growth rate and the chlorophyll content of *Brassica napus* [79].

Iron (Fe) is also essential in minute amounts in plants in order to maintain proper growth and development. Fe availability can lead to the destruction of key metabolic and physiological processes, leading to reduced plant yield [80].

Manganese (Mn) acts as a cofactor for many enzymes, providing the ability to withstand various environmental constraints. Furthermore, Mn is imperative for photosynthesis and the synthesis of ATP, chlorophyll, fatty acids, proteins, and secondary metabolites such as lignin and flavonoids [80]. The effects of laboratory-prepared NPs, such as Zn, Cu, Mn, and iron oxide, on the germination of lettuce (*Lactuca sativa*) seeds were assessed at low concentrations (<50 mg/L), and the study revealed that CuO NPs were similar to Zn NPs. In addition, the growth of lettuce seedlings was enhanced by MnO and FeO NPs [81].

### 5.1. Silica (Si/SiO/SiO_2_/Silicon)

Silica NPs promote seedling growth and activate antioxidant enzymes in order to resist biotic and abiotic stresses [82,83]. The two major hurdles in agriculture are salinity and drought, which reduce the crop production. Under salt stress, Si NPs increase the potassium uptake and reduce the sodium uptake [84]. The advantages of silica and silicon NPs for plant growth and development can practically combat unfavourable climate changes, such as high temperatures and erratic rainfall (because silica can mitigate the evaporation of water from the soil surface).

### 5.2. Titanium

TiO_2_ has been utilised commercially as a pigment in paints and sunscreens, owing to its photocatalytic activity [85]. TiO_2_ is primarily used in NP studies that are related to plant growth, plant responses to seed germination, pest management, pesticide degradation, superior water purification, and sensor technology, in order to detect pesticide residues, because it converts light energy into electrical or chemical energy under sunlight. TiO_2_ NPs have been used for numerous crops, such as spinach [86], watermelon [87], tomato [88], bean [89], millet [79], lettuce [90], *Lemna minor*, and wheat [91]. These studies using TiO_2_ NPs concluded that they increase the germination rate, the yield/biomass, the chlorophyll content, the photosynthetic activity, and the nutrient content. Moreover, physiological activities were also improved due to an increase in the nitrogen metabolism and RuBisCO. However, plant responses toward TiO_2_ NPs depend on the plant type, the nanoscale properties, and the exposure concentration [92]. Furthermore, plants that are exposed to TiO_2_ NPs grow faster by capturing more sunlight [93].

### 5.3. Carbon-Based Nanomaterials

Carbon is the main constituent of all living organisms and forms the fundamental structure of various biomolecules (proteins, carbohydrates, and fats) through atomic bonds. Plants use carbon for growth and to provide food to other living organisms. Manure, or decomposing plant biomass, is the carbon source that is used by farmers to nourish soil. Regardless of the carbon source (organic carbon from the soil or atmospheric carbon dioxide), plant growth and carbon are interrelated. Engineered carbon nanostructures have attracted significant interest because of their stability and physicochemical characteristics. Plants uptake CNTs that influence their growth and development [94]. An array of studies on carbon nanomaterials/structures in plants have shown positive results and have concluded that they increase the root length and plant biomass and stimulate seed germination [95].

## 6. Nanobiofertilizers

**Figure 2:**
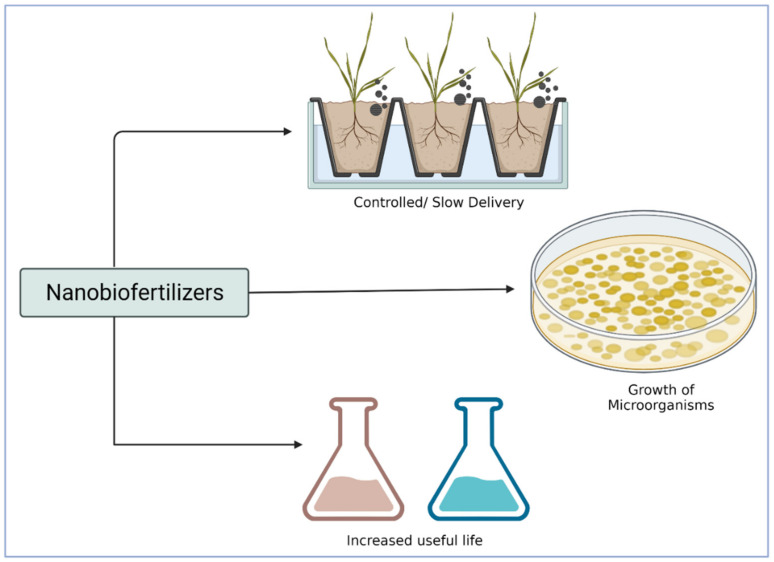
The functional mechanism of nanobiofertilizers in plants. An illustration depicting the major advantages of nanobiofertilizers.

Biofertilizers are formulations that enhance the soil productivity with a combination of one or more microorganisms by fixing atmospheric nitrogen, synthesising the growth-promoting substances, and solubilising phosphorus [96,97]. Therefore, a combination of biofertilizers and nanostructures can be referred to as nanobiofertilizers [98,99]. The interaction between NPs and microorganisms, the longevity of biofertilizers, and their transport are the three most important aspects of nanobiofertilizers (Figure 2). Although three categories of microorganisms—plant growth-promoting rhizobacteria (PGPR), nitrogen-fixing rhizobacteria, and arbuscular mycorrhizal fungi—may be used for nanobiofertilizers, only PGPR have been used as biofertilizers in the majority of cases [99]. A positive effect was documented in a previous study [100], which revealed the interaction between gold NPs and PGPR. However, silver NPs in biofertilizers have an adverse effect on the biological functions of microorganisms [101]. The stability of biofertilizers in terms of tolerance to desiccation, heat, UV inactivation, and overcoming shelf life is enhanced.

## 7. Role of Nanofertilizers in Hydroponically Grown Crops

Currently, many plants and crops are cultivated using hydroponics because of a lack of space. The use of nanofertilizers for the growth of hydroponically grown crops is not unheard of. Hydroponically grown plants show traces of magnetic NPs in their roots, stems, and leaves, whereas plants that are grown in soil or sand show no such signals, indicating no particle uptake. Although nanoscale Zn and ZnO decreased seed germination in rye grass and corn, research on zucchini seed germination and root growth in a hydroponic solution containing ZnO NPs showed no negative response. Hydroponically grown soybean plants consumed ZnO NPs in their Zn^2+^ oxidation state. Hydroponic cultures of velvet mesquite were used by Hernandez-Viezcas et al. [102] in order to examine the effects of 10 nm-sized ZnO NPs (500–4000 mg L^−1^) under stress. The plants showed increased specific activity of catalase and peroxidase enzymes in response to NP exposure, with no adverse effects, such as chlorosis, necrosis, stunting, or wilting, indicating a high tolerance level toward ZnO NPs [102]. Marchiol et al. prepared hydroxyapatite (Ca_10_(PO_4_)_6_(OH)_2_) NPs for use as nanofertilizers for the growth of *Solanum lycopersicum* L. and found that increased concentrations of NPs had no effect on germination, however, root elongation was significantly stimulated [103]. Al-Juthery et al. [48] conducted experiments using nano-silicon fertilizer (NS), nano-complete fertilizer (NC), and Atonikin (A) in a controlled environment in order to determine which one would be most effective in maximising hydroponically grown barley fodder. The statistical analysis using the least significant difference test showed that compared to the untreated plants, the dry matter content, the crude protein, the crude fat, the crude fibre, the acid detergent fibre, and the neutral detergent fibre were significantly higher in the plants that were grown in NS+A+NC hydroponic solution [48]. Cucumber plants that were grown in hydroponics with N-doped ACP NPs showed equal root and shoot biomass development without nitrogen depletion in in vivo experiments with half of the absolute N content of the standard urea treatment. The sustainability of the practical use of N-doped ACP as a nanofertilizer is supported by its high nitrogen utilisation efficiency (up to 69%) and cost-effective fabrication process [104]. Salt stress has emerged as a major threat to agricultural production and food supply because of climate change and rapid population growth. Managing salt stress through the use of nanofertilizers in farming is an exciting new area of research. The effects of two alfalfa (*Medicago sativa* L.) genotypes (susceptible: Bulldog 505, and tolerant: Mesa-Sirsa) that were cultivated with different salt concentrations (6 and 10 dSm^−1^) in a split-split configuration were studied using a hydroponic experiment. Nano-K_2_SO_4_ improved several plant parameters, including the shoot dry weight, the plant height, the flower count, the tiller count, the root length, the root fresh weight, and the root dry weight, for all of the salt concentrations. Both of the genotypes showed increased electrolyte leakage and relative water content after the addition of nano-K_2_SO_4_, although the salt amounts were different [105].

## 8. Systematic Study

The effect of nanomaterials on plants depends on the interaction between the intrinsic and extrinsic properties of NPs. This interaction is one of the reasons why the same class of NPs shows contrasting results, as reported in the literature. For example, corn seeds that were exposed to TiO_2_ NPs showed delayed germination [106], whereas wheat seed germination was improved, and rice seed germination was not affected by the same treatment [87]. There is no doubt that plant nutrients are supplied to the soil in a controlled and precise manner by nanofertilizers; however, little information is available about the fate of these nanomaterials in the soil. Nanomaterials can form aggregates in soil, and the behaviour of NPs in these aggregates is largely affected by the soil porosity, the soil granularity, the organic content of the soil, the soil biota, the pH, and other soil conditions. The accumulation of large-sized aggregates over time may hinder the movement of nutrients and minerals, affecting their stability. In addition, nano-toxicity may also arise in the soil and harm the plants in the long run [107,108].

## 9. Uptake, Translocation, Delivery, and Biodistribution of NPs

The direct manufacture or production of nanoscale materials is becoming crucial for application in various emerging fields, such as life sciences, agriculture, ecology, and medical science. Nanomaterials are frequently synthesised by aerosol-based processes [109], with a range of elements of single, doped, and composite NPs, metal, metal alloys, metal oxides, polymers, and “wet” methods such as sol-gel, homogenous precipitation, biosynthesis using an enzyme and protein template, hydrothermal, and reversed micelles methods [35]. Nanofertilizers require a product that is capable of producing large-scale particles at an affordable cost.

There are generally three ways to deliver agrochemicals—foliar spray, seed treatment, and soil amendment. The exposure to NPs, when they are mixed with the soil (direct exposure), has numerous consequences, as localised concentrations show a greater increase compared to that by foliar spray (indirect exposure). Furthermore, high exposure concentrations may affect microbial communities in the soil or the rhizosphere [110], which could restrict the uptake of particles by plants [111]. Nutrient and pesticide applications through the leaves have been performed for many years [112]. Nutrient deficiency in the soil is treated by soil application or seed treatment by the fertilizer, whereas deficiency symptoms that are exhibited by plants are treated by foliar (aerosol) application [113]. For foliar applications, a higher leaf area index and a low exposure dose with potentially multiple applications and weather-based applications are essential in order to avoid nutrient loss [112]. The aerosols of engineered NPs may be harmful when they are exposed to the air or when they are inhaled by humans or other animals. However, the particle concentration in the atmosphere, the weather conditions, the exposure concentration, and the physicochemical properties of the particles may determine the intensity of their effects [114]. In order to ensure the safe foliar application of nanofertilizers, suitable personal shield equipment, such as masks, gloves, and eye-protective glasses, should be used [115]. The properties of nanofertilizers are important for foliar delivery [116]. Additionally, it was demonstrated that foliar application of iron and magnesium NPs to peas (*Vigna unguiculata*) had a significant positive impact on the plant growth and development [117]. Similar experiments have been performed with aerosol-mediated NPs in watermelons and tomatoes [118].

## 10. Controlled Release and Targeted Delivery of Nanoscale Material to Plants

NPs have been widely employed in nanomedicine as therapeutic agents and drug delivery systems [119]. Nanomaterials can be modified for precise applications in plants [120]. For instance, Torney et al. [121] used silica NPs in order to deliver a gene and its chemical inducer to intact tobacco leaves and isolated plant cells (protoplasts). The use of nanomaterials for the delivery of vital nutrients, herbicides, and genetic elements will provide new possibilities for the agricultural revolution [78].

## 11. Transport Models for Nutrient Uptake in Plants

**Figure 3:**
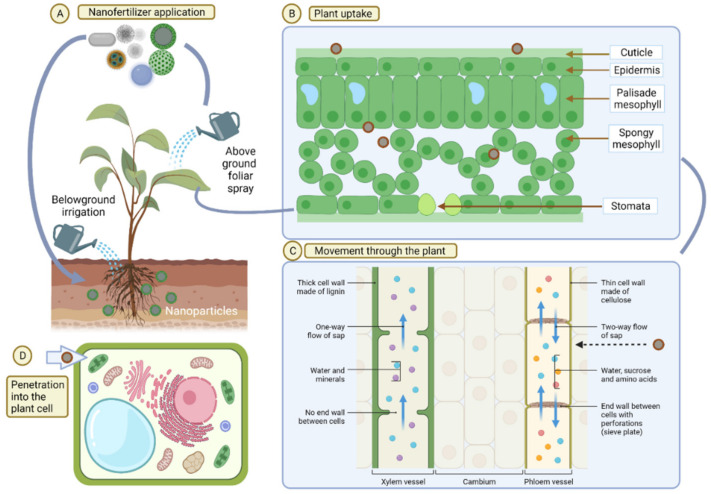
Application, uptake, translocation, and biodistribution of nanofertilizers inside the plant cells.

Various models have been used to address nutrient uptake and transportation in various plant parts. The process of uptake typically entails the passage of nutrients from the soil toward the root surface, the transport of ions via the membranes of root surface cells, the radial transport of ions into the root xylem vessels, the transport in the xylem, and the distribution of ions in the aboveground parts of the plant (Figure 3) [122]. Recent studies have focused on determining the total nutrient uptake over time, as well as the nutrient uptake of a particular root and its growth rate.

## 12. Advantages of Nanofertilizers over Conventional Chemical Fertilizers

Nanofertilizers are non-toxic and are less harmful to humans and the environment than the conventional fertilizers. In addition, they increase the soil fertility, the yield, and the crop quality; minimise the costs; and optimise the profit [123]. Carmona et al. recently reported that the functionalisation of amorphous calcium phosphate nanofertilizers post-synthesis (instead of its single-pot synthesis) has substantially reduced the manufacturing costs, and this new method is now being utilized for the large-scale production of nanofertilizers in order to help small-scale farmers and plant breeders [104]. Chemical fertilizers are employed by farmers in large quantities in order to promote crop output because they are synthetic, meaning that they are made of non-organic cultivated ingredients [124]. Chemical fertilizers function more quickly than organic fertilizers because they instantly dissolve in water, they can be found in granular or liquid forms, and they are less expensive. However, because some insoluble chemicals are present in P fertilizers, including mono-ammonium phosphate, diammonium phosphate, and triple superphosphate, they do not readily dissolve in water [125]. Organic substances, including animal dung, bird droppings, food waste, and sewage sludge, are broken down by soil microorganisms, thus releasing the vital nutrients. Because it enhances the soil texture, increases the activity of the soil bacteria and fungi, and stores water for longer periods, this natural fertilizer is more environmentally friendly [126]. N, K, and P, which shield plants from pests and diseases, comprise the majority of the nutrients that are released from this material. The biggest drawback of this type of fertilizer, compared with chemical fertilizers, is the slower release of nutrients. Biofertilizers are excellent substitutes for synthetic fertilizers because they improve the soil quality and contain the vital nutrients that are necessary for plant fertility and productivity. They are also inexpensive, renewable, and environmentally friendly [127]. Several microorganisms, such as *Azotobacter*, *Anabaena*, and *Rhizobium*, that are involved in N fixation, and *Pseudomonas* spp., which serves as phosphate-solubilising bacteria, act as biofertilizers by assisting plants in nutrient uptake and absorption [128]. These microorganisms generate several kinds of bioactive compounds, organic acids, vitamins, growth hormones, and antagonistic compounds, thus protecting plants from diseases, in addition to fixing nitrogen, and increasing the availability of nutrients to the plants.

## 13. Limitations and Drawbacks of Nanofertilizer Use

Although the use of NPs as fertilizers to promote agricultural production and to increase the availability of plant nutrients is gaining interest, there are some toxicity-related risks. In addition, gaps exist in the research, the legislation, and sufficient monitoring, which hamper the large-scale application of nanofertilizers. These small particles can penetrate biological systems more deeply and pose great potential dangers; hence, the toxicity, the safety, and the effects of NPs on the environment are still unknown [129]. Chemical and physical procedures yield NPs that are more dangerous than those that are produced by biological approaches. Moreover, the organic NPs are less toxic to the soil microorganisms than the metal and metal oxide NPs [130]. Although NPs are being utilised to deliver nutrients to plants, the nano-toxicity remains a matter of concern for both humans and the environment [60,108]; hence, extensive research is required on the toxicity of biologically manufactured NPs. Currently, there is no suitable legislation or risk management system to monitor the use of nanofertilizers for sustainable crop production. Notably, nanofertilizers are not being produced or made available in quantities that are required to meet the demands of their wide-scale application in plant nutrition [131]. The higher cost of nanofertilizers, compared to that of the conventional fertilizers, is a concern when it comes to their wide application under different pedo-climatic conditions across the earth, which is a big hurdle, coupled with the fact that there is a lack of standardisation and recognised formulation of these fertilizers, resulting in contrasting effects on the same plants across different areas [128,132].

Most importantly, a bitter truth is that many products are currently marketed that are not actually “nano”-fertilizers and are of “micron” size, which further suggests a lack of monitoring of nanofertilizers [19,133]. These particles have severe toxic effects on the long-term persistence in plant systems.

## 14. The Existing Challenges and Future Prospects for the Use of Nanofertilizers

Agrochemical use in sustainable agriculture must be minimised, and creating a plant nutrient system that is effective and not harmful to the environment is crucial for this effort. Most tropical and subtropical soils are acidic, are frequently substantially deficient in P, and have a high potential for phosphate sorption [134]. Consequently, unique and cutting-edge methods to increase crop productivity are being developed using nanotechnological and nanoengineering techniques [135]. Enabling a sustainable agricultural supplement, especially in developing nations with abundant native phosphate rock resources, where phosphate rocks are processed industrially, and the proper use of phosphate rocks as P sources can contribute to global development [136]. Other sustainable options for managing P consumption include optimising the land use, minimising erosion, preserving the soil quality, structuring better fertilizer replacement techniques, enhancing the fertilizer recommendations, selecting the most suitable crop genotypes, and exporting manure. In order to adjust the amount of product that is supplied to the crops or individual plants, instruments are required that can monitor crop requirements and evaluate the inter- and intra-crop demand variability [137]. However, owing to the constrained reserves, it is anticipated that the prices of inputs for agriculture, such as fertilizers and pesticides, will surge dramatically. The use of nanomaterials to deliver vital nutrients to crops via nanotechnology-based delivery systems is still new and requires further research [138]. There are some potential future directions to consider when organising future research on nano-based fertilizers—(a) N- and P-nanofertilizers may receive more attention because of their high application rate and availability concerns. The investigation may compare Ca-NPs from CaCO_3_ with those from other Ca sources, such as CaSO_4_ or CaCl_2_, or it may compare several nutrients, such as Ca- and N-NPs, using soluble Ca (NO_3_)_2_ as a control; (b) in the case of micronutrients, an in-depth study may be performed in order to clarify the impact of the variables influencing their availability in field conditions. Comparing their value to traditional fertilizers for biofortification and application techniques (using fertigation) with commercially available micronutrient fertilizers is another option. Because the primary problem with these kinds of fertilizers is nano-toxicity, the optimal dosage for each crop (without any adverse effects) should be determined; (c) currently, there is no conclusive evidence to support the ability of nanocarrier-based fertilizers to boost FUE through improved nutrient transfer into the plant tissues or cells or to lower the environmental hazards that are related to the use of conventional fertilizers. There is a chance to discover specific nanomaterials, such as SiO_2_-NPs, Fe_2_O_3_-NPs, and CNTs, which are less expensive and more effective than the previously accessible carriers, such as zeolite. These developments would aid in the gradual absorption of active substances and would minimise the quantity of inputs that are needed and the waste that is generated. Moreover, the design of nanocarriers may enable them to act as anchors for the roots of plants or for the organic matter and soil structure around them. Understanding the chemical and conformational interactions between the delivery nanoscale structures and the targeted soil structures and materials is necessary in order to achieve this; (d) it has been conclusively established that different NPs/nanomaterials at high concentrations are harmful to plant growth, which ultimately depends on their particle size. Hence, a checkpoint may be developed in future investigations in order to determine the required concentrations and to further understand the mechanisms underlying the increased growth and productivity of crop plants due to the application of nanofertilizers. However, nanotechnology may be able to control the sustainable release of agrochemicals and the site-targeted delivery of macromolecules, such as fertilizers and pesticides, thus promoting plant disease resistance, efficient nutrient use, and enhanced plant growth. In addition, other modernised techniques, such as satellite-based navigation systems, including remote sensing, global positioning systems, geographic information systems, and information technologies, can be employed in order to ensure that crops and soils receive precise nutrients for optimum productivity [139,140].

## 15. Conclusions

As an innovation in material design and consumer product development, a customised nanofertilizer was created. Although the application of these approaches in agriculture is still in its infancy, it can change agricultural systems, especially with regard to the problems with manure application [141]. By minimising fertilizer expenses and emission hazards, the use of diverse nanofertilizers can have a remarkable effect on crop productivity. Owing to their increased solubility, reactivity, and ability to penetrate the cuticle, nanofertilizers provide targeted distribution and controlled release [142]. Moreover, by reducing abiotic stress and heavy metal toxicity, nanofertilizers can enhance the crop growth, the yield, the quality, and the nutrient use efficiency. However, attention is being drawn to the risks that are associated with consuming and using the technology in limited ways, rather than its benefits and efficacy [143].

With recent developments in nanotechnology, unique NPs and nanomaterials have been developed that can boost crop growth and production while also acting as carriers for macro- and micronutrients. According to the evidence that has been gathered, the impact of NPs varies with the type of plant and is influenced by their manner of application, size, shape, and concentration. Once the optimal dosage and plant requirements for nanofertilizers are established, the crop production can be greatly increased. Future crop plants could benefit greatly from greener nanonutrition, especially in light of the known nanotoxicological effects of nanomaterials and NPs. Therefore, green nanomaterials/NPs may potentially be used as a nutrient source for crops, which would significantly contribute to more environmentally friendly nanonutrition.

## Figures and Tables

**Table 1:** Effects of foliar application of nanofertilizers in different crops and plants.

Sl. No.	Nanomaterials	Crop Species	Conc. Used	Mode of Application	Duration of Treatments	Responses	Reference
1	ZnO	*Cyamopsis tetragonoloba*	10 mg/L	Foliar spray	4–6 weeks	Increases in biomass accumulation and nutrient concentration, and enhancements to growth physiology	[36]
2	CuO	*Solanum lycopersicum*	150–340 µg/mL	Foliar application	11 days	Eliminated the spread of disease	[43]
3	ZnO and Fe_3_O_4_	*Moringa peregrina*	30, 60, and 90 mg/L	Foliar application	Watered every 3 days	Salinity levels reduced the growth parameters significantly	[44]
4	Zn, Fe and NPK	Chickpea	20 L/plot at each stage	Foliar application	First Spraying at 4 to 6 leaf stage, second spraying at 30 days, and third spraying during pod filing	There was a significant increase in both biological and seed output	[45]
5	Zn and B NPs	*Punica granatum*	0, 60, and 120 mg ZnL^−1^	Foliar application	Once every season and one week before the first full bloom	Increases in pomegranate fruit yield	[46]
6	Al_2_O_3_ NPs	*Solanum lycopersicum*	400 mg/L	Foliar application	20 days	Effectively counteract Fusarium as a biocontrol agent	[47]
7	N, P and NPK NPs	*Triticum aestivum*		Foliar application		Significant changes in plant growth parameters like shoot length, root length and others	[48]
8	NPK NPs	Wheat grains	500, 60, and 400 ppm	Foliar spray	Treatment after 21 days of the date of planting	Significant increase in total saccharide content of wheat grains	[49]
9	ZnO	*Coffea arabica*	15 mg/L	Foliar spray	40–45 days	Acceleration of net photosynthesis and increased biomass production	[50]
10	AgNPs	*Vigna unguiculata*	30–90 µg/mL	Foiar application	4–7 days	Growth inhibition of X. axonopodis pv. malvacearum and other harmful bacteria	[51]
11	TiO_2_ and SiO_2_	*Oryza sativa*	20 and 30 mg/L	Foliar application	55 days	Better development and Cd translocation inhibition	[52]

## Data Availability

Not applicable.
